# High seroprevalence of *Toxoplasma gondii* infection in inmates: A case control study in Durango City, Mexico

**DOI:** 10.1556/EuJMI.4.2014.1.7

**Published:** 2014-03-14

**Authors:** C. Alvarado-Esquivel, J. Hernández-Tinoco, L. F. Sánchez-Anguiano, A. Ramos-Nevárez, S. M. Cerrillo-Soto, L. Sáenz-Soto, O. Liesenfeld

**Affiliations:** ^1^Biomedical Research Laboratory, Faculty of Medicine and Nutrition, Juárez University of Durango State, Avenida UniversidadS/N. 34000 DurangoMexico; ^2^Institute for Scientific Research “Dr. Roberto Rivera-Damm”, Juárez University of Durango State, Avenida UniversidadS/N. 34000 DurangoMexico; ^2^Institute for Scientific Research “Dr. Roberto Rivera-Damm”, Juárez University of Durango State, Avenida UniversidadS/N. 34000 DurangoMexico; ^3^Clínica de Medicina Familiar, Instituto de Seguridad y Servicios Sociales de los Trabajadores del Estado, Predio CanoasS/N. 34079 DurangoMexico; ^3^Clínica de Medicina Familiar, Instituto de Seguridad y Servicios Sociales de los Trabajadores del Estado, Predio CanoasS/N. 34079 DurangoMexico; ^3^Clínica de Medicina Familiar, Instituto de Seguridad y Servicios Sociales de los Trabajadores del Estado, Predio CanoasS/N. 34079 DurangoMexico; ^4^Institute for Microbiology and Hygiene, Campus Benjamin Franklin, Charité Medical SchoolHindenburgdamm 27, D-12203 BerlinGermany; ^1^Biomedical Research Laboratory, Faculty of Medicine and Nutrition, Juárez University of Durango State, Avenida UniversidadS/N. 34000 DurangoMexico; ^2^Institute for Scientific Research “Dr. Roberto Rivera-Damm”, Juárez University of Durango State, Avenida UniversidadS/N. 34000 DurangoMexico; ^3^Clínica de Medicina Familiar, Instituto de Seguridad y Servicios Sociales de los Trabajadores del Estado, Predio CanoasS/N. 34079 DurangoMexico; ^4^Institute for Microbiology and Hygiene, Campus Benjamin Franklin, Charité Medical SchoolHindenburgdamm 27, D-12203 BerlinGermany

**Keywords:** epidemiology, infection, inmates, Mexico, seroprevalence, *Toxoplasma gondii*

## Abstract

**Purpose:**

The seroprevalence of infection with the parasite *Toxoplasma gondii* and the association with risk factors has not been determined in inmates. Through a case-control study, 166 inmates from a state correctional facility in Durango City, Mexico and 166 age- and gender-matched non-incarcerated subjects were examined for the presence of anti-*T. gondii* IgG and IgM antibodies using enzyme-linked immunoassays.

**Results:**

Seroprevalence of anti-*T. gondii* IgG antibodies was higher in inmates (35, 21.1%) than in controls (14, 8.4%) (OR = 2.90; 95% CI: 1.43–5.94; *P* = 0.001). Anti-*T. gondii* IgM antibodies were detected in two (1.2%) inmates and in seven (4.2%) controls (*P* = 0.17). Multivariate analysis of socio-demographic, incarceration, and behavioral characteristics of inmates revealed that *T. gondii* seropositivity was associated with being born out of Durango State (OR = 3.91; 95% CI: 1.29–11.79; *P* = 0.01). In addition, *T. gondii* seroprevalence was higher (*P* = 0.03) in inmates that had suffered from injuries (17/56: 30.4%) than those without such history (18/110: 16.4%).

**Conclusions:**

The seroprevalence of *T. gondii* infection in inmates in Durango City is higher than the seroprevalences found in the general population in the same city, indicating that inmates may represent a new risk group for *T. gondii* infection. Further research on *T. gondii* infection in inmates is needed.

## Introduction

*Toxoplasma gondii* infection is a common parasitic infection present in about a third of the humanity [1]. Most *T. gondii* infections are clinically unapparent but some infected individuals develop lymph node, eye, and central nervous system diseases [2]. Infections with *T. gondii* in immunocompromised patients may lead to a life-threatening disease [3]. In addition, primary infections with *T. gondii* in pregnant women represent a risk for congenital disease [4]. *T. gondii* is a food-borne protozoan [5]. There are two major routes of *T. gondii* infection: by ingesting food or water contaminated with oocysts shed by cats and by ingestion of raw or undercooked meat containing tissue cysts [2]. Transmission of *T. gondii* may also occur by blood transfusion [6] or organ transplantation [7].

Inmates are of epidemiological importance because they have an increased prevalence of a number of infectious diseases, including infections with human immunodeficiency virus [8, 9], hepatitis B virus [8], hepatitis C virus [10], and tuberculosis [11]. Furthermore, food-borne infections caused by bacteria and viruses have been described in inmates [12–14]. In a recent study, a high seroprevalence of *Helicobacter pylori* exposure was found in inmates [15]. In addition, the high seroprevalence of HIV among inmates is of concern because co-infection of HIV and *T. gondii* represents a risk for a life-threatening disease of the central nervous system. However, the prevalence of infection with *T. gondii* in inmates and the association with risk factors for infection has not been investigated yet. Therefore, we sought to determine the seroprevalence of *T. gondii* infection in inmates in Durango City, Mexico. In addition, we determined the association of seropositivity to *T. gondii* with the socio-demographic, clinical, incarceration, and behavioral characteristics of the inmates.

## Materials and methods

### Study design and study populations

We performed a case control seroprevalence study using serum samples from previous *T. gondii* [16] and viral hepatitis [17] surveys. One hundred and sixty-six inmates (cases) and 166 controls were examined for the presence of anti-*T. gondii* IgG and IgM antibodies. Cases and controls were randomly selected. Inclusion criteria for the inmates were incarceration for at least 6 months in the state correctional in Durango City, aged 18 years and older, any gender, and who accepted to participate in the study. Inmates included in the study were 18–73 (mean = 33.34 ± 10.80) years old, 127 were males and 39 were females. Inmates were living under crowding conditions. The correctional facility houses inmates that have committed a range of crimes. However, the types of crimes committed were not asked of the inmates in the present survey. Controls were subjects without incarceration from the general population in the same Durango, City. Controls were matched with cases by age and gender. Controls were 18–74 (mean = 33.33 ± 10.81) years old, 127 were males and 39 were females. Age was comparable between cases and controls (*P* = 0.99).

### Socio-demographic, clinical, incarceration, and behavioral data

We used archival questionnaires with socio-demographic, clinical, incarceration, and behavioral characteristics of the inmates. Socio-demographic data included age, gender, birthplace, residence, marital status, occupation, and socioeconomic level. The clinical status of inmates was obtained. History of blood transfusions, surgeries, and injuries in inmates was obtained. In addition, in female inmates, obstetric history was obtained. Incarceration characteristics examined included number of incarcerations, jail section, and duration of current incarceration. Behavioral data obtained were foreign travel, alcoholism, drug abuse, presence of piercing and tattoos, and sexual behavior.

### Serological detection of  *T. gondii* antibodies

Sera from inmates and controls were analyzed by qualitative and quantitative methods for anti-*T. gondii* IgG antibodies with the commercially available enzyme immunoassay kit “*Toxoplasma* IgG” (Diagnostic Automation Inc., Calabasas, CA, USA). Specific anti-*T. gondii* IgG antibody levels were expressed as International Units (IU)/ml, and a result equal to or greater than 8 IU/ml was considered positive. Sera positive for anti-*T. gondii* IgG antibodies were further analyzed for anti-*T. gondii* IgM antibodies by the commercially available enzyme immunoassay “*Toxoplasma* IgM” kit (Diagnostic Automation Inc., Calabasas, CA, USA). All tests were performed following the manufacturer’s instructions. Positive and negative controls were included in each assay.

### Statistical analysis

We performed the statistical analyses with the aid of the following software: Microsoft Excel 2010, Epi Info version 3.5.4 (Centers for Disease Control and Prevention: http://wwwn.cdc.gov/epiinfo/) and SPSS version 15.0 (SPSS Inc. Chicago, Illinois). Comparison of age between cases and controls was performed by the Student’s *t* test. We used the Pearson’s chi-square test and the Fisher exact test (when values were less than 5) to determine the association of *T. gondii* seropositivity with the inmate characteristics. For multivariate analysis, only inmates characteristics with a *P* value ≤ 0.25 obtained in the bivariate analysis were included. Multivariate analysis was performed using logistic regression analysis with the Enter method. Odds ratios (OR) and 95% confidence intervals (CI) were calculated, and statistical significance was set at *P* value < 0.05.

### Ethics statement

This study was approved by the Ethical Committee of the Institute for Security and Social Services of the State Workers in Durango City. Archival sera and data from previous surveys were examined in the present study. In the previous studies, the purpose and procedures of the surveys were explained to all participants, and a written informed consent was obtained from each participant.

## Results

Anti-*T. gondii* IgG antibodies were detected in 35 (21.1%) of 166 inmates and in 14 (8.4%) of 166 controls. Seroprevalence of anti-*T. gondii* IgG antibodies was significantly higher in inmates than in controls (OR = 2.90; 95% CI: 1.43–5.94; *P* = 0.001).Of the 35 anti-*T. gondii* IgG positive inmates, 22 (13.3%) had anti-*T. gondii* IgG antibody levels higher than 150 IU/ml, 4 (2.4%) between 100 and 150 IU/ml, and 9 (5.4%) between 8 and 99 IU/‌ml. In comparison, of the 14 anti-*T. gondii* IgG positive controls, 12 (7.2%) had anti-*T. gondii* IgG antibody levels higher than 150 IU/ml, 1 (0.6%) between 100 and 150 IU/‌ml, and 1 (0.6%) between 8 and 99 IU/ml. High (>150 IU/ml) levels of anti-*T. gondii* IgG antibodies were comparable among inmates and controls (*P* = 0.07). Seroprevalence of anti-*T. gondii* IgM antibodies did not differ in inmates (2, 1.2%) and controls (7, 4.2%) (*P* = 0.17). Both inmates positive for anti-*T. gondii* IgM antibodies had high (>150 IU/ml) levels of anti-*T. gondii* IgG antibodies, and they had been in jail for 10 months.

Anti-*T. gondii* IgG antibodies were detected in 6 (15.4%) of 39 female inmates and in 1 (2.6%) of 39 female controls (*P* = 0.10). While anti-*T. gondii* IgG antibodies were detected in 29 (22.8%) of 127 male inmates and in 13 (10.2%) of 127 male controls. Seroprevalence of anti-*T. gondii* IgG antibodies was significantly higher in male inmates than in male controls (OR = 2.59; 95% CI: 1.27–5.26; *P* = 0.006). The prevalence of high (>150 IU/‌ml) anti-*T. gondii* IgG antibody levels was similar in male (18/127: 14.2%) and female (4/39: 10.3%) inmates (*P* = 0.78).

With respect to socio-demographic and incarceration characteristics *(Table 1)*, four characteristics had *P* values ≤ 0.25: birth place (*P* = 0.0001), residence before incarceration (*P* = 0.003), marital status (*P* = 0.003), and number of incarcerations (*P* = 0.25). Other socio-demographic and incarceration characteristics in inmates including age, gender, occupation, socioeconomic status, jail section, and duration of incarcerations had *P* values > 0.25.

**Table 1.** Socio-demographic and incarceration characteristics of inmates and seroprevalence of *T. gondii* infection

Characteristic	Subjects tested^a^	Prevalence of *T. gondii* infection		*P* value
	No.	No.	%	
Gender				
Male	127	29	22.8	0.31
Female	39	6	15.4	

Age groups (years)				
30 or younger	83	20	24.1	0.6
31–50	69	12	17.4	
>50	14	3	21.4	

Birth place				
Durango State	119	16	13.4	0.0001
Other Mexican state or abroad	47	19	40.4	

Residence				
Durango State	133	22	16.5	0.003
Other Mexican State	33	13	39.4	

Marital status				
Single	45	4	8.9	0.003
Married	70	12	17.1	
Divorced	4	0	0.0	
Living together	40	15	37.5	
Widowed	6	3	50.0	

Occupation				
Laborer^b^	151	33	21.9	0.73
Non-laborer^c^	15	2	13.3	

Socio-economic level				
Low	105	20	19.0	0.69
Medium	51	12	23.5	
High	3	1	33.3	

Jail section				
A	23	5	21.7	0.51
B	3	0	0.0	
C	38	6	15.8	
D	40	7	17.5	
E	62	17	27.4	

Number of incarcerations				
One	128	30	23.4	0.25
Two or more	38	5	13.2	

Duration (years) of current incarceration				
0.5–1	45	9	20.0	0.88
1.1–2	56	12	21.4	
2.1–3	23	6	26.1	
3.1–5	26	6	23.1	
More than 5	16	2	12.5	

^a^Subjects with available data ^b^Laborer: Agriculture, construction worker, business, driver, factory worker, other ^c^Non-laborer: student or housekeeping				

Of the clinical characteristics, the seroprevalence of *T. gondii* infection in healthy inmates (22.5%) was comparable with that (18.2%) found in ill inmates (*P* = 0.51). Seroprevalence of *T. gondii* infection was similar (*P* = 0.33) in inmates with blood transfusions (5/16: 31.3%) than those without blood transfusions (30/150: 20%). The frequency of *T. gondii* infection was similar (*P* = 1.0) in inmates with history of surgeries (17/83: 20.5%) than those without surgeries (18/83: 21.7%). In contrast, seroprevalence of *T. gondii* infection was higher (*P* = 0.03) in inmates that had suffered from injuries (17/56: 30.4%) than those without such history (18/110: 16.4%). In women, none of the obstetric characteristics, including pregnancies, deliveries, cesarean sections, and abortions, was associated with *T. gondii* seroprevalence.

Concerning the behavioral characteristics of the inmates examined *(Table 2)*, only two variables had *P* values ≤ 0.25 in the bivariate analysis: alcoholism (*P* = 0.12), and no use of condom (*P* = 0.09). Multivariate analysis of socio-demographic, incarceration and behavioral characteristics of inmates that had *P* values ≤ 0.25 in the bivariate analysis revealed that only one characteristic was associated with *T. gondii* seropositivity: being born out of Durango State (OR = 3.91; 95% CI: 1.29–11.79; *P* = 0.01).

**Table 2.** Bivariate analysis of selected behavioral characteristics in inmates and *T. gondii* seroprevalence

Characteristic	Subjects tested^a^	Prevalence of *T. gondii* infection		*P* value
	No.	No.	%	
National trips				
Yes	79	14	17.7	0.39
No	82	19	23.2	

Traveled abroad				
Yes	59	13	22	0.82
No	107	22	20.6	

Drug abuse				
Yes	66	15	22.7	0.69
No	99	20	20.2	

Alcoholism				
Yes	104	26	25	0.12
No	62	9	14.5	

Tattoos				
Yes	52	10	19.2	0.69
No	114	25	21.9	

Piercing				
Yes	59	13	22	0.82
No	107	22	20.6	

Sexual promiscuity				
Yes	61	11	18	0.4
No	102	24	23.5	

Condom use				
Yes	42	5	11.9	0.09
No	120	29	24.2	

Homosexuality				
Yes	8	2	25	0.67
No	158	33	20.9	

Bisexuality				
Yes	2	0	0	1
No	163	35	21.5	

^a^Subjects with available data				

## Discussion

The impact of infection on inmates has been studied for a number of viral and bacterial infections but not for the protozoan parasite *T. gondii*. In a study of 279 autopsies of prison inmates and non-incarcerated patients with AIDS in Texas, USA, researchers found *T. gondii* encephalitis in nine cases [18]. However, the report did not mention whether such nine cases were observed in inmates or non-incarcerated patients. Since there is not any previous report on the seroprevalence of *T. gondii* infection in inmates, the present study was performed to investigate the seroprevalence and correlates of infection with *T. gondii* in inmates in Durango, Mexico.

Crime has been associated with mental disorders, i.e., violent behavior of patients suffering from schizophrenic and bipolar disorder is an important public health problem [19]. Of interest, schizophrenia has been linked to homicidal behavior [20], and infection with *T. gondii* appears to be associated with schizophrenics [21–23] and other psychiatric disorders [24, 25]. Thus, one can hypothesize that on one hand infection with *T. gondii* may trigger mental disorders that subsequently lead to behavior changes, crime, and incarceration; on the other hand, incarceration may increase the likelihood of infection with the parasite due to exposure to particular risk factors.

Interestingly, we found that the prevalence of *T. gondii* exposure was significantly higher in inmates than in controls. In addition, the high seroprevalence of *T. gondii* exposure found in inmates (21.1%) is one of the highest seroprevalences reported in population groups in Durango City until now. Seroprevalences found in inmates are as high as the 20% seroprevalence of *T. gondii* exposure reported in patients suffering from schizophrenia [22] and the 21.1% seroprevalence found in waste pickers in Durango City [26]. The seroprevalence found in inmates is also higher than the 6.1–13.3% seroprevalences found in healthy and ill populations in Durango City, including blood donors [27], general population [16], patients with visual impairment [28], liver diseases [29], and people occupationally exposed to raw meat [30] and unwashed fruits and vegetables [31]. The seroprevalence found in inmates is comparable with the weighted mean national seroprevalence of *T. gondii* infection (19.27%) reported in Mexico [32]. It is not clear why inmates had a higher seroprevalence of *T. gondii* exposure than controls. Inmates were confined to the correctional facility, and cats were not observed in the correctional facility. It is not clear whether infection with *T. gondii* was obtained before or during incarceration. Only two inmates had anti-*T. gondii* IgM antibodies and their duration in jail had been 10 months. We were unable to obtain information about eating habits known to be associated with *T. gondii* infection in inmates. Remarkably, seroprevalence of *T. gondii* infection in inmates was high even in the youngest individuals, and there was no increase with age as reported in general population [16]. Regression analysis showed that being born outside of Durango State was associated with *T. gondii* seropositivity. Therefore, such characteristic may have contributed for the high seroprevalence of *T. gondii* exposure in inmates. Previous studies in Durango have shown that seroprevalence of *T. gondii* exposure was higher in persons born in Mexican States other than Durango State than those born in Durango, including general population [16], elderly people [33], and patients suffering from hearing and visual impairments, cancer, human immunodeficiency virus infection, or undergoing hemodialysis [28]. It is likely that inmates born outside Durango State have different behavioral risk for *T. gondii* infection than inmates born in Durango. Intriguingly, regression analysis showed that *T. gondii* was similar in inmates regardless their residence either in Durango or outside Durango. It is not clear why inmates borne outside Durango have a higher seroprevalence than those born in Durango. Unknown risk factors for *T. gondii* infection are present in inmates born outside Durango State. We were unable to examine other putative risk factors for *T. gondii* infection, including contact with cats and meat consumption in inmates, because we studied archival data from a hepatitis viruses survey that contained only data related with parenteral or sexual transmission of hepatitis viruses. We cannot rule out the contributing role of food or water in *T. gondii* exposure in inmates. However, information about the source of food was not provided. It is unclear whether the risk of eating contaminated food or water is higher in correctional facilities than elsewhere. Further studies to examine behavioral factors associated with *T. gondii* exposure in inmates are needed. Results of the present study raise a number of questions: 1) Are there any mental disorders associated with *T. gondii* infection in inmates that have contributed to commitment of crimes? 2) Is there any type of crime associated with infection with *T. gondii?* 3) Did inmates obtain the infection before incarceration or during their stay in jail? 4) Was any food related with infection with *T. gondii* in inmates? Additional studies should be conducted to address these issues.

We conclude that the seroprevalence of *T. gondii* exposure in inmates is higher than those found in non-incarcerated controls and in the great majority of population groups studied in the same city. Therefore, results indicate that inmates are a new risk group for *T. gondii* exposure. Results warrant for further research on *T. gondii* infection in inmates.
